# *In silico* discovery of novel transcription factors regulated by mTOR-pathway activities

**DOI:** 10.3389/fcell.2014.00023

**Published:** 2014-06-03

**Authors:** Agnieszka Jablonska, Natalia Polouliakh

**Affiliations:** ^1^Faculty of Biotechnology and Food Sciences, Lodz University of TechnologyLodz, Poland; ^2^Fundamental Research Laboratories, Sony Computer Science Laboratories Inc.Tokyo, Japan; ^3^Systems Biology InstituteTokyo, Japan; ^4^Graduate School of Medicine, Yokohama City UniversityYokohama, Japan

**Keywords:** mTOR pathway, orthologous genes, transcription factors, bioinformatic tools and databases, cell signaling

## Abstract

The mammalian target of rapamycine (mTOR) pathway is a key regulator of cellular growth, development, and ageing, and unraveling its control is essential for understanding life and death of biological organisms. A motif-discovery workbench including nine tools was used to identify transcription factors involved in five basic (*Insulin, MAPK, VEGF, Hypoxia*, and *mTOR core*) activities of the mTOR pathway. Discovered transcription factors are classified as “process-specific” or “pathway-ubiquitous” with highlights toward their regulating/regulated activities within the mTOR pathway. Our transcription regulation results will facilitate further research on investigating the control mechanism in mTOR pathway.

## Introduction

Computational analysis of transcription regulation of biological pathways is a challenging task because a single pathway may be interconnected to multiple upstream and downstream pathways and, as a consequence, might be regulated by various groups of transcription factors (Schmelzle and Hall, 2000; Hay and Sonenberg, 2004; Inoki and Guan, 2006; Polouliakh et al., 2006, 2009; Laplante and Sabatini, 2009; Caron et al., 2010). Because the transcriptional level is a fundamental mechanism that is well conserved in all cellular systems, a cross-species comparison approach should increase the confidence of the analysis. A further way to reduce false positives is to combine the consistent part of the results obtained on multiple analytic tools.

In this work, a transcription regulation network of orthologous human-mouse mTOR (mammalian target of rapamycin) pathway genes was investigated by using nine public motif-discovery tools published in the last decade. TOR was first discovered in yeast (Heitman et al., 1991), then identified as evolutionarily conserved (Brown et al., 1994; Sabatini et al., 1994), and finally functionally characterized in fungi yeast (Lorberg and Hall, 2004), animals (Hall, 2008), and plants (Deprost et al., 2007). It has a high molecular weight and several distinctive domains. In particular, mTOR consists of 2549 amino acids and possesses a kinase domain at the C terminal, which displays homology to the phosphatidylinositol 3-kinase (PI3K) catalytic domain. PIK–related kinases are responsible for numerous cellular processes, e.g., cell growth and development, DNA-damage detection, and telomere maintenance (Schmelzle and Hall, 2000; Hay and Sonenberg, 2004). Because of its structure, mTOR can create two functional protein complexes with two other proteins: one with MLST8 and RICTOR and one with MLST and RPTOR. Since it can form such structures, mTOR has to be analyzed in the context of a wider signaling network (Inoki et al., 2005).

An mTOR pathway is an important regulator of embryogenesis, angiogenesis, adipogenesis, autophagy, and cell cycle. It can be divided into several branches responsible for actin and microtubule organization, protein and lipid synthesis, cell growth and cell differentiation, and autophagy (Laplante and Sabatini, 2009). In addition, the involvement of an mTOR pathway in the aging process and pathological changes such as tumor formation, cancer, insulin resistance, and diabetes has been pointed out by many researches (Laplante and Sabatini, 2009; Stanfel et al., 2009; Caron et al., 2010). What's more, disruption of genes from mTOR pathway (e.g., TSC1, TSC2, and TOR) results in early embryonic death both in *Drosophilla* and mammals, suggesting that TOR plays a crucial role in the development (Kobayashi et al., 2001; Murakami et al., 2004; Inoki et al., 2005). In humans, cell hypertrophy, hyper-function and hyperplasia, typically associated with activation of mTOR, contribute to diseases associated with aging, such as myocardial infarctions and strokes, osteoporosis, cancer, autoimmune disease, arthritis, obesity, diabetes, macula-degeneration, and Alzheimer's and Parkinson's diseases (Blagosklonny, 2006; Johnson et al., 2013; O'Neill and Hardie, 2013).

An mTOR pathway is regulated by various signals. It can be activated by availability of amino acids and nutrients, presence of growth factors and hormones, and it may be halted by the action of such factors as energy stress, hypoxia, and osmotic changes (Inoki et al., 2005; Inoki and Guan, 2006; Caron et al., 2010). It is also regulated by the level of gene expression, which is controlled by transcription factors (TFs) within the pathway.

When investigating transcription regulation, it is necessary to consider both the comparatively short promoter regions in the proximity of a transcriptional start site (TSS), where the polymerase II transcription initiation complex forms, and distantly located enhancer regions that may interact with one or more promoter sites of neighboring genes (Serizawa et al., 2003) and whose distance from the TSS can exceed 100 kb.

Given that scientific tools can be difficult to install, it is particularly helpful for biologists to be able to use these tools through a web-user interface. Our investigations were focused on tools available online and showed that nine out of thirty-two tested online motif discovery programs are easy and suitable for the analysis, while others should be handled offline or require additional data management.

As a result of our study with selected tools, 61 transcription factors for human and 50 transcription factors for mouse were identified, and 24 and 13 transcription factors, respectively, were then selected for the discussion on their activity in mTOR pathway. Twenty-one transcription factors between human and mouse were orthologous, and the 13 most frequent of them are discussed in this paper.

Transcription factors *regulating* an mTOR pathway and transcription factors *regulated* by an mTOR pathway are indicated separately. We believe that the created transcription regulation map can extend the knowledge from already verified transcription factors to the new hypothesis of transcription regulation of an mTOR pathway including feedback loops. Our guidance on a bunch of recent motif-discovery tools will be helpful for a large scope of audience and should trigger further discussion on optimization of tools and analysis workflow (Ghosh et al., 2011).

## Materials and methods

### mTOR pathway gene dataset

The mTOR human pathway was downloaded from the KEGG database (map04150) and included 49 human genes with 43 orthologous partners in mouse. Promoter regions of 5000 base pairs upstream and 500 base pairs downstream with their representative transcriptional start sites (TSS) were retrieved from the DBTSS database (Yamashita et al., 2010).

### Motif discovery tools and motif selection

Thirty-one non-commercial online motif-discovery programs, which are already popular for the analysis of biological data or were newly developed in the last decade, were investigated. In our set of tools we included programs that could analysis orthologous promoters (e.g., Pscan, TransFind), programs with diverse methodologies (e.g., MEME_Chip, TFM-explorer, Pscan) and programs that link the results to the expression data to augment probability likelihood of predicted motifs (e.g., MEME_Chip, PASTAA, TRAP). Next, each of selected program had to fulfill the following four characteristics: (1) capability to handle a large data size (300 Kb) and return results in less than 24 h; (2) report motif results for each DNA sequence or report motif results across the whole dataset; (3) execute a software operation without manual parameter adjustment; and (4) annotate resulted motifs in currently available databases.

Five main programs, which report identified motifs in each sequence, namely, Pscan (Zambelli et al., 2009), PASTAA (Roider et al., 2009), MEME_Chip (Machanick and Bailey, 2011), TFM-Explorer (Defrance and Touzet, 2006), and XXmotif (Luehr et al., 2012) were selected, and four supportive programs, which report motifs over a whole dataset, namely, Cscan (Zambelli et al., 2012), LocaMo (Vandenbon et al., 2013), TransFind (Kielbasa et al., 2010), and TRAP (Roider et al., 2007), were selected.

All programs were executed in their default parameter setting. Table 1 lists features of the nine programs used for the analysis.

**Table 1:**
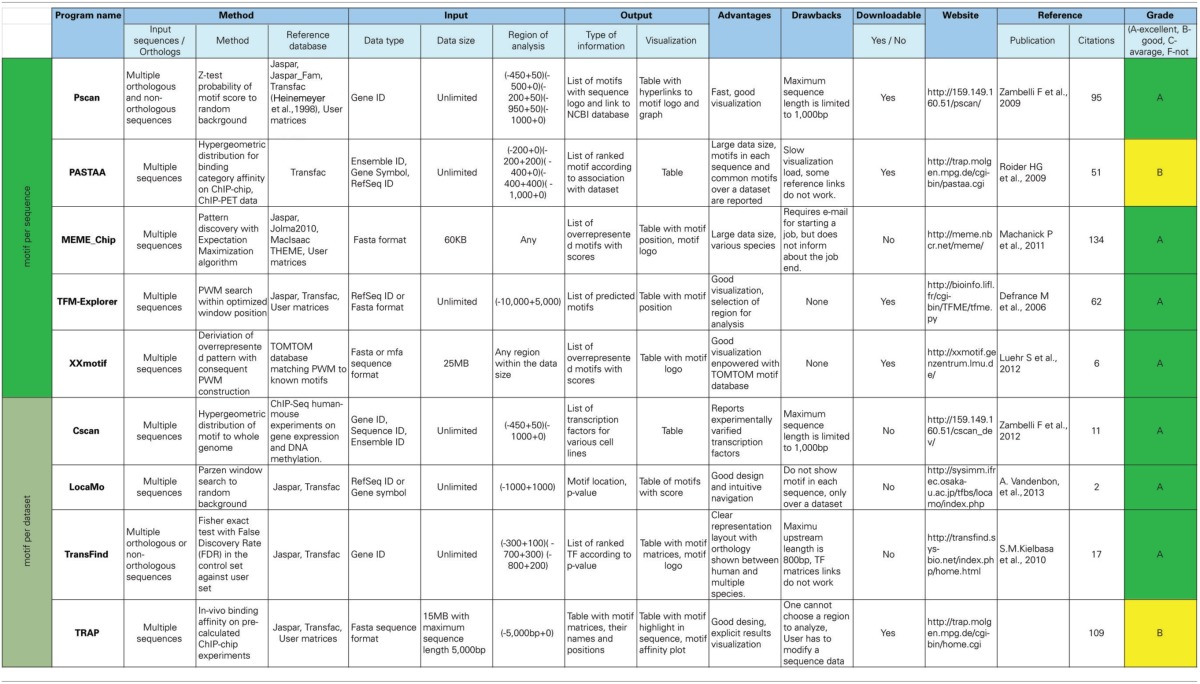
**Programs used in this study**.

*The table includes following columns: “Method” column specifies the orthologous/non-orthologous approach, algorithm/statistics used, and the database used for motif annotation; “Input” describes size, format, and promoter region available for analysis; “Output” introduces results format and visualization capacity of the software. The next columns are “Advantages” and “Drawbacks” with useful/bulky features highlighted from the biologist's viewpoint. The next few columns post website information, publication citations, and availability of the software for downloading. The last column entitled “Grade” ranks programs according their suitability impact on our analysis. The leftmost column separates two types of programs: those that output found motifs per sequence; and those that output motifs over a dataset, classified as main and supportive in our analysis, respectively (see Methods). Grades are assigned as “A” (very good) and “B” (good)*.

Twenty-two online programs that were investigated and found unsuitable for our analysis are listed as follows: BioProspector (Liu et al., 2001), CompareProspector (Liu et al., 2004), CompMoby (Chaivorapol et al., 2008), ConTra (Broos et al., 2011;) ConSite (Sandelin et al., 2004), Improbizer (Ao et al., 2004), Gibbs Motif Sampler (Thompson et al., 2003), Lasagna (Lee and Huang, 2013), MDScan (Liu et al., 2002), Melina (Poluliakh et al., 2003), MEME (Bailey et al., 2009), Mobyle @Pasteur tfscan (Néron et al., 2009), MotifSearch (Dinh et al., 2011), MotifViz (Fu et al., 2004), MotifVoter (Wijaya et al., 2008), PhyloGibbs (Siddharthan et al., 2005), RSAT consensus, rVista (Loots and Ovcharenko, 2004), SeSiMCMC (Favorov et al., 2005), SCOPE (Carlson et al., 2007), WebMOTIFS (Romer et al., 2007), and YMF (Sinha and Tompa, 2003).

Initial motifs were collected in accordance with two conditions: first, each of the five main selected programs should identify a motif and, second, some supportive programs should also find the same motif. Details of collected motifs are represented in Supporting Figure 1, together with their target genes and software programs used.

To find the most co-regulated genes within the dataset, the average number of transcription factors per gene was calculated by dividing the total number of TFBS motifs found by PScan, PASTAA, MEME_Chip, TFM-Explorer, and XXmotif in a human dataset (1,295), by the total number of genes in the human dataset (49) that resulted in 26.43 (*SD* ± 7.87) transcription-factor motifs per gene, shown above the red horizontal line in Supporting Figure 1A, hereafter called a *gene threshold* (Gene_TH). This resulted in 24 genes, possessing more than 26 predicted motifs in their promoters for human, including their multiple occurrences.

The top 24 out of 61 (40%) human transcription factors found in the promoters of genes above the *gene threshold* were taken and denoted as those above a *transcription factor threshold* (TF_TH). This selection showed that at least half of the 24 genes possessed each of the 24 above-selected transcription-factor motifs in their promoters. The top-occurring transcription factors was SP1 with its highest frequency and top occurrence of seven copies in the RPS6KA3 gene, and the last-occurring transcription factor was ARNT found in 12 genes with single occurrence (Supporting Figure 1A). This 24-by-24 matrix is named an *intersection zone*.

**Figure 1:**
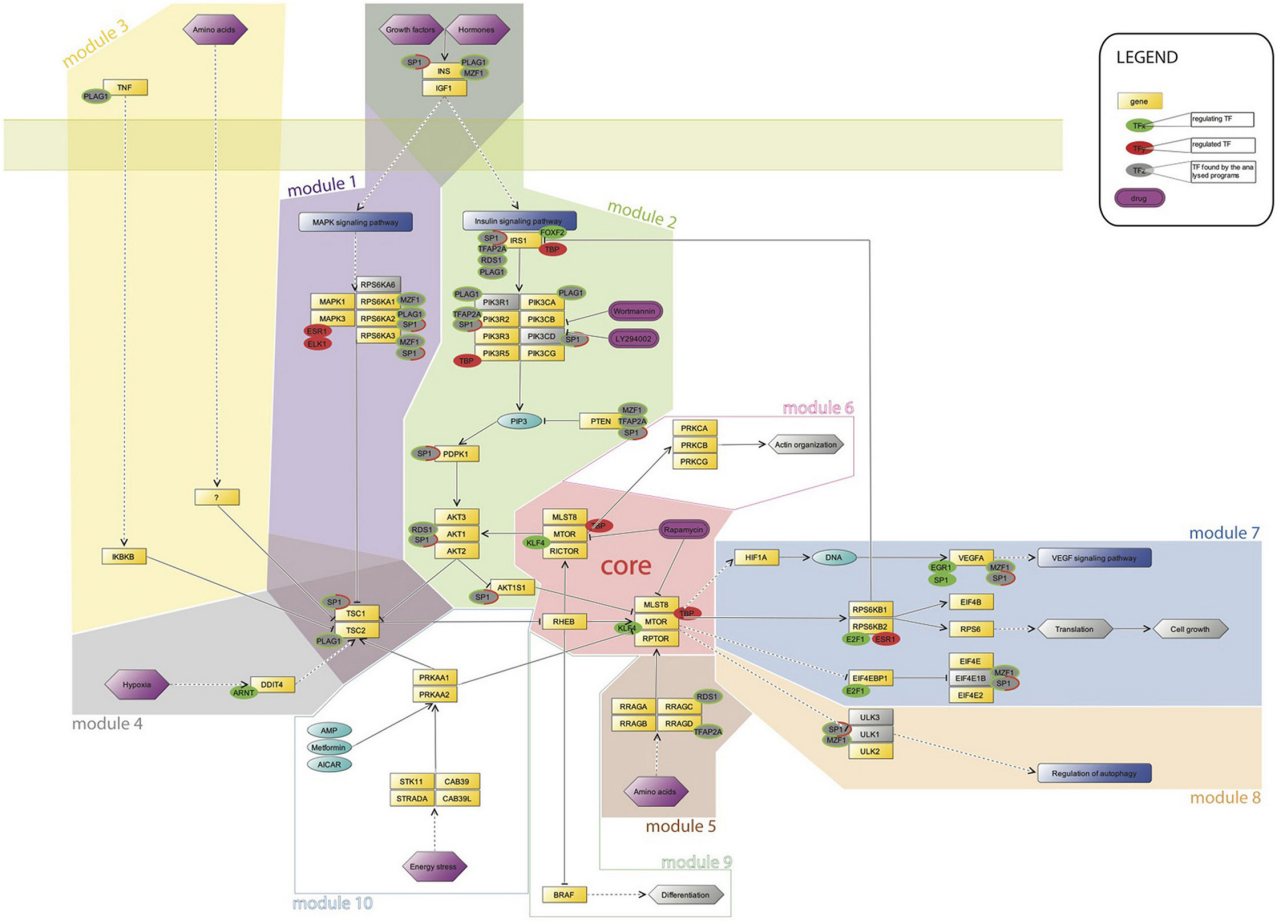
**mTOR map with transcription factors identified in this study**. TFs shown in green and red are those already assigned to the mTOR pathway and re-confirmed in our study. The former are those with a regulating role in the mTOR pathway, and the latter are those regulated by the mTOR pathway genes. Gray circles are newly identified TFs with rims colored according to their regulating or regulated status in mTOR. Genes without orthologs for mice are shown in gray squares. Modules are shown upon the basic knowledge on mTOR-pathway-regulation workflow with assigned respective TFs detected in our study.

The intersection zone was used to produce an mTOR map by CellDesigner4.3 (Funahashi et al., 2003) with those TFs having multiple copies in the promoters or those TFs already mentioned in the literature in relation to mTOR (Figure 1). The transcription-regulation map was divided into an *mTOR core* and ten *modules* for convenience to point out to the transcription factors involved in different activities of the mTOR pathway. Modules were then classified into pre-core transcriptional modules and post-core transcriptional modules according to their place within the pathway.

Transcription factors were classified as those that regulate the mTOR pathway and those that are regulated by the mTOR pathway, representing them in green and/or red circles on the map in Figure 1. Newly identified TFs are shown in gray circles with a respective color rim depending on their role in the mTOR pathway. Human genes without orthologs in mice are shown in gray squares on the map (Figure 1). The intersection zone for human is extended to mouse data to see commonalities between the two species (Supporting Figures 1A,B).

## Results

### mTOR-pathway transcription factors

Transcription factors (TF) and genes allocated in the intersection zone (see Methods) were selected as candidates for regulating an mTOR pathway. The initial identified number of TFs was 61 for human and 50 for mouse with 21 orthologs (Figure 1 and Supporting Figure 1). Nine transcription factors identified in our study were already assigned to the activities of an mTOR pathway, but their target genes were not specified. Those TFs are following: SP1, KLF4, TBP, ELK1, ESR1, E2F1, FOXF2, EGR1, and ARNT, shown with respective green or red colors in Figure 1 according to their regulating or regulated role for in regard to an mTOR pathway. TFs having orthologous binding motifs in mouse are marked in Supporting Figure 1C with asterisks. Previously unknown transcription factors identified in our study in mTOR-pathway genes are shown in gray on the map in Figure 1. We found particularly interesting those TFs with number of binding sites exceeding three occurrences per gene, i.e., high-affinity TFs, and those appearing in more than a half of the promoters of genes in the dataset, i.e., in our *intersection zone* (see Methods). Activities of various modules with assigned transcription factors are discussed in the next sub-sections.

### mTOR core, pre-core, post-core transcriptional modules

The pre-core mTOR modules are listed as following: *module 1*—induced by growth factors and hormones possessing MAPK cascade and RPS6K genes; *module 2*—activated by growth factors and hormones and involved in insulin signaling pathway associated with PIK3 genes and AKT genes; *module 3*—regulated by extracellular amino acids and TNF; *module 4*—possessing hypoxia dependent genes; *module 5*—connected with the balance of amino acids inside the cell and related to the RRAG gene group; *module 9*—linked with cell differentiation; *module 10*—containing energy-stress-related genes such as CAB3, STRADA, and STK11 as well as genes influenced by drug administration.

Post-core transcriptional modules are listed as follows: *module 6*—associated with actin organization; *module 7*—connected to VEGF pathway conducting cell development and growth; and *module 8*—modulating autophagy. As an mTOR pathway *core*, an RHEB gene and two blocks of genes with MTOR-RICTOR and MTOR-RPTOR interacting pairs, respectively, is considered. *Modules 3*, *6*, *8*, and *9* are shown on the map in Figure 1, but they are not discussed in this paper because no reliable motifs were identified in our study.

### Module one of MAPK-signaling pathway and module two of insulin-signaling pathway

In *module 1, which* embodies MAPK signaling pathway, five transcription factors were identified. Two of them, ESR1 and ELK1, are already known, and three newly identified in our study are SP1, MZF1 (myeloid zinc finger 1), and PLAG1 (Pleiomorphic adenoma gene 1 protein).

Both ELK1 (an ETS domain-containing protein) and ESR1 (a nuclear steroid receptor) interact with the MAPK3 gene (Polouliakh et al., 2006). ELK1 is a nuclear target for a MAPK cascade (Uht et al., 2007). ESR1 is a homodimer composed of several domains crucial for hormone binding and form heterodimers with ESR2 (estrogen receptor 2). In the mTOR pathway, ESR1 is inhibited by phosphophorylation regulated by RPS6KB1 (S6K1) from a VEGF signaling pathway and a MAPK pathway (Yamnik and Holz, 2010) (*module 7*). Inhibition of ESR1 affects activation of S6K1/2 and stimulates phosphorylation of S6K1/2 and 4EBP in the presence of Leucine. ELK1 and ESR1 are regulated by a mTOR MAPK signaling pathway and a VEGF angiogenesis signaling pathway with crosstalk.

SP1 is the most abundant in *modules 1* and *2* as well as in other parts of the mTOR pathway. SP1 is a zinc finger protein and binds to motifs rich in GC content in various promoter regions to sustain an open chromatin structure of DNA. SP1 regulates various processes of mTOR pathway, which are cell differentiation, cell growth, apoptosis, immune responses, response to DNA damage, and chromatin remodeling. The allocations of binding motifs for SP1 within promoters of genes in the mTOR pathway suggest the strong possibility of both regulating and regulated statuses of SP1 in the mTOR pathway.

Discovering SP1 in EIF4E1B in module 7 of the mTOR pathway proves the above-mentioned suggestion. EIF4E1B is an inhibitor of eukaryotic translation initiation factor EIF4E—a group of genes involved in mRNA activation, protein synthesis, and cell development (Li et al., 2012). Moreover, in the mTOR pathway, SP1 interacts with hypoxia inducible factor HIF1, transactivating vascular endothelial growth factor (VEGF) regulating the VEGF pathway (*module 7*) and influencing autophagy (*module 8*) (Alam et al., 2004).

A myeloid-zinc-finger (MZF1) TF function, which is a granulocyte precursor in bone marrow, in the mTOR pathway is not yet discovered. Found in the multiple promoters of module 1 it can be presumed that MZF1 changes the transcription inside the pathway by influencing INS (dependent on growth factors and hormones) or by regulating the MAPK cascade and RPS6K gene (*module 1*). Moreover, MZF1 is also involved in activating or deactivating the PTEN gene, which is known as a tumor suppressor (*module 2*). MZF1 binding motifs were also found in VEGFA promoter, suggesting regulation of the VEGF pathway (*module 7*), in E1F4E1B promoter implying involvement in cell development (*module 7*), and in ULK1 promoter indicating connection with autophagy process in mTOR (*module 8*) [27]. Accordingly, MZF1 is a ubiquitous plays an important role in regulation of the whole mTOR pathway.

The third transcription factor contributing largely to the pre-core modules one and two is PLAG1 (pleiomorphic adenoma gene 1 protein). It regulates a range of mTOR processes involved in cell proliferation, organ growth, and apoptosis. In our research, it is found in the promoters of INS, RPS6KA2 (*module 1*), IRS1, PIK3R1, PIK3CA (*module 2*), TNF (*module 3*), and TSC2 (*module 4*). Akhtar et al. (2012) suggest that PLAG1 is overexpressed in cancer cells when it binds to IGF2 promoter. Because of the close proximity of the IRS1 and IGF genes, PLAG1 may also regulate IRS1 as it was identified in our research.

Pre-core *modules 1* (MAPK signaling) and *2* (Insulin signaling) share SP1, MZF1, and PLAG1 transcription factors. Apart from it, *module 2* has FOXF2 (forkhead box F2) and TBP (TATA-binding protein). FOXF2 and TBP are already known in the context of *co*-regulation with MTOR, RDS1, (adenine-repressible gene) and TFAP2A (transcription factor AP-2 alpha) (Westergren, 2010).

FOXF2 is classified as TF, which regulates the mTOR pathway. The increased amount of FOXF2 inhibits the activity of IRS1 (insulin receptor substrate 1), leading to inhibition of the whole mTOR pathway via a negative-feedback loop (Westergren, 2010). FOXF2 interacts with another transcription factor TBP (TATA-binding protein), and in our research, both of them were found in close proximity to IRS1. TBP additionally interacts with SP1 and E2F1 transcription factors found in the *intersection zone* of mostly interconnected genes in our study (Supporting Figure 1A). In *module 2*, TBP is regulated by PI3K genes, and in the mTOR *core*, TBP is regulated by the MTOR gene, thus, playing a vital role in recruiting Polymerase I and modulating activity of Polymerase III (Cianfrocco et al., 2013).

In consideration of the locations of TFAP2A binding motifs, it is noteworthy that TFAP2A can regulate the mTOR pathway and, in particular, its *core* path. A TFAP2A binding motif was found in the promoters of IRS1 and PIK3R2 genes of an insulin signaling pathway (*module 2*), a PTEN gene, which is an inhibitor of the mTOR pathway thought the PIP3 (*module 2*) and RRAGD genes (*module 5*). TFAP2A is thus involved in processes of organ development and negative cell proliferation at the same time. One of the probable scenarios concerning TFAP2A is that by inhibition of IRS1, PIK3R2 (*module 2*), and RRAGD (*module 5*) genes, TFAP2A stimulates the PTEN transcription immobilizing mTOR pathway and negative feedback loop may exist between insulin-signaling *module 2* and amino-acid balance *module 5*. Surprisingly, a TF characteristic for yeast RDS1 (an adenine-repressible gene), responsible for the regulation of transcription from RNA polymerase II, is identified in the human data set. RDS1 binding motif is found in the promoters of IRS1, AKT1 (threonine-protein kinase) genes of pre-core *module 2*, and the RRAGC (Ras-related GTP binding C) gene of post-core *module 5*. It is difficult to assess RDS1 functions in an mTOR pathway due to the fact that it is only been found in yeast to date. However, in yeast organisms, it is responsible for transcription regulation and response to xenobiotic stimulus. In human organisms, its function may be very similar to the function in yeast, and human ortholog might be identified in the future.

RDS1 can influence the mTOR pathway from two sides: first, being regulated by growth factors and hormones (IRS1 and AKT1 in *module 2*) and, second, being regulated by the availability of amino acids or generally intracellular xenobiotics (RRAGC *in module 5*). The above-described finding on RDS1 activity indicates the existence of crosstalk between module 2 (insulin signaling) and module 5 (amino-acid balance within a cell) transcription regulation and existence of a negative feedback loop between them. This is a biologically reasonable finding as insulin-control amino-acid metabolism is tightly related to amino-acid-uptake balance.

### Module four regulates hypoxia

ARNT (aryl-hydrocarbon receptor nuclear translocator 3, HIF-1β) is one that regulates gene expression in hypoxia *module 4*. It encodes a protein that is crucial for complex formation with the ligand-bound aryl hydrocarbon receptor (AHR) and proper functioning of this receptor. Current knowledge indicates that the HIF1- and/or HIF2-mediated hypoxia responses can be oncogenic as well as tumor suppressive (Pawlis and Hu, 2013). ARNT induces activation of REDD1 (DDIT4) by creating a dimer with HIF1, thereby inhibiting TORC1 and affecting the TSC2-depending mechanism (Kapahi et al., 2010). ARNT interacts with ESR1, co-activating its transcription (Endler et al., 2004), and with SP1, creating a reconstituent complex (Mulero-Navarro et al., 2006) with it. SP1 itself is able to bind the Ah receptor and down-regulates its expression in leukemia cells (Mulero-Navarro et al., 2006).

### *Core* module of mTOR

In the core of mTOR pathway KLF4 (Kruppel-like factor 4) inhibits the MTOR gene and has an anti-proliferative effect on the whole mTOR pathway (Wang et al., 2012), thereby promoting self-renewal and precluding differentiation.

On the contrary, MEF2A (myocyte specific enhancer factor) positively regulates genes of the mTOR/S6K pathway, so it belongs both to the mTOR *core* and *module 7* (VEGF pathway) (Pereira et al., 2009; Yin et al., 2012), promoting cell growth and differentiation. However, the MEF2 family is also responsible for cardiac hypertrophy and failure (Pereira et al., 2009), and its overexpression leads to cardiac dysfunction.

### Module seven of VEGF pathway

*Module 7* is associated with VEGF (vascular endothelial growth factor) pathway-advancing cell growth, organ development, and protein formation. This transcriptional module possesses four transcription factors, namely, SP1, ESR1, E2F1, and EGR1, with three first TFs tightly associated with the activities of the mTOR pathway.

EGR1 (early-growth-response protein 1), which is *module 7* specific TF, directs cell differentiation. It was discovered that its level in a prostate cancer was raised, showing a pro-oncogenic character; however, in other tumor types (skin tumor, fibrosarcoma, and glioblastoma), EGR1 exhibits features of a tumor suppressor by activating p53 and PTEN to halt the transcription of other genes in the mTOR pathway (Zheng et al., 2009). Moreover, it was discovered that the knockdown of EGR1 affects VEGFA, thereby affecting the VEGF pathway and mitogenesis (Abdel-Malak et al., 2009).

An E2F group affects activation of RPS6KB1 (S6K) and stimulates phosphorylation of S6K and 4EBP in the presence of Leucine. E2F1 integrates cell division (as well as cell growth) and induces an apoptotic response (Real et al., 2011). In tumor cells, it can activate EGR1 to endorse cell survival. Moreover, in tumor cells, by up-regulating the production of EGR1, epidermal growth factor (EGFR), platelet-derived growth factor (PDGFRA), and insulin-like growth factor II (IGF2BP2), E2F1 can activate the phosphoinositide-3-kinase/Akt (PIK3CA/AKT) pathway in a way to inhibit drug-induced apoptosis (Zheng et al., 2009).

## Discussion

Transcription factors selected (24 genes) from the results analysis where those identified by main and supportive motifs discovery tools used in our study (Supporting Figure 1). Other transcription factors that less frequently appear can be seen in the process-module-assigned fashion in Supporting Figures 1A,B for human and mouse, respectively. Transcription factors (24) are classified into two groups: first, those expressed in the specific module of mTOR and second, those commonly identified in various modules.

The first group of TFs includes ESR1, ELK1 (MAPK signaling, *module 1*), FOXF2 (Insulin signaling, *module 2*), KLF4 (*mTOR core*), ARNT (Hypoxia, *module 4*), E2F1, and EGR1 (VEGF signaling, *module 7*). The second group includes SP1, MZF1, PLAG1, TFAP1A, TBP, and RDS1. Among member of the second group, MZF1, PLAG1, TFAP1A, and RDS are newly identified. Other transcription factors identified in the promoters of genes in our analysis are EGR2, EGR3, YY1, SREBF1, SREBF2, ELF1, and ARID3A. They are less frequently share by the promoters in our dataset but they are well-known to be involved in the mTOR pathway regulation (YY1, SREBFs).

One-third (21) of the human transcription factors were found to be conserved in the case of a mouse. Among them most confident are SP1, TFAP2A, MZF1, EGR3, KLF4, USF1, KLF9, and ARNT (Supporting Figure 1B). They are transcription factors having multiple copies in gene promoters and/or shared by many genes within the dataset. Cross-species-conserved TFs identified corroborate the consistency of our methodology. However, a part of frequently appearing mouse TFs, such as HSF1, CDC5L, HES6, and FOXL1, were not identified in orthologous human promoters. This might be regarding to the difference in the transcription regulation between two species.

The results described in the paper indicate that the proper selection of online motif-discovery tools without parameters tuning is feasible to bring accurate results for the discovery transcription regulation on medium size data. However the aim to reduce false positives might result in the omitting low sensitivity-degenerate motifs (Polouliakh et al., 2005). Creation of sophisticated analytic workflow might be warranted to cope with large-scale sequence data for *de novo* motif discovery.

## Author contributions

Agnieszka Jablonksa analyzed data and wrote the manuscript. Natalia Polouliakh discussed the research and wrote the manuscript.

### Conflict of interest statement

The authors declare that the research was conducted in the absence of any commercial or financial relationships that could be construed as a potential conflict of interest.

## Supplementary material

Supporting Figure 1 **Transcription-factor (TF) binding motifs in the mTOR pathway identified in human (A) and mouse (B) orthologous datasets**. The intensity of the blue color reflects frequency of motif occurrence per gene. The colors above transcription-factor names point to those supportive programs (Cscan, LocaMo, TransFind, and TRAP), which also identified a motif after it was detected by the five main programs (MEME_Chip, PASTAA, Pscan, TFM-Explorer, and XXmotif). Transcription factors were sorted according to the sum of occurrences in the whole data set. The red horizontal line in the human-case results highlights 24 genes with average number of motifs per gene higher than 26 (Gene threshold, Gene_TH), and the vertical red line shows the top-24 transcription factors (TF_TH) identified as the most significant in our study. Sub-table **(B)** shows mouse transcription factors and genes in the same order with the human ones and extended red lines from human data in order to depict similarities between two species. Sub-table **(C)** includes description of transcription factors and estimated positions of their motifs in human promoters based on the results of the five main programs (MEME_Chip, PASTAA, Pscan, TFM-Explorer, and XXmotif) and asterisks point to human-mouse orthologous TFs.
